# Epidermal Junctions and Skin Inflammation

**DOI:** 10.3390/jcm11195915

**Published:** 2022-10-07

**Authors:** Elena Donetti

**Affiliations:** Department of Biomedical Sciences for Health, Università degli Studi di Milano, 20133 Milan, Italy; elena.donetti@unimi.it

Epidermal junctions help to preserve cutaneous homeostasis and, consequently, protect the body against a wide range of environmental stresses. In the highly dynamic and finely tuned epithelial compartment of the skin, i.e., the epidermis, the most represented junctions are desmosomes (DSMs), adherens junctions (AJs), and tight junctions (TJs), which are interspersed with each other on the keratinocyte cell membrane [1]. DSMs and AJs are anchoring structures that are present throughout the epidermal compartment, linking the intermediate filaments and the actin cytoskeleton to the plasma membrane, respectively. The former are composed of homo/heterodimers of transmembrane proteins belonging to the desmocollin and desmoglein families [2], and the latter are characterized by the expression of E-cadherin and beta-catenin [3]. Hemidesmosomes, which are confined in the basal layer, fix cells to the basement membrane at the epidermal–dermal junction, whilst corneodesmosomes are specifically localized in the uppermost differentiated stratum corneum. TJs, on the other hand, are restricted to the upper granular layer, although molecular components, such as claudin 1 and Zonula Occludens (ZO)-1, are also expressed in less differentiated layers, suggesting that they can have further roles beyond intercellular connection [4]. Recently, there has been growing interest with reference to the involvement of epithelial junctions in cellular processes other than mechanical anchorage, such as stress response, autophagy, and lysosome and proteasome-mediated degradation [1].

TJs, together with the stratum corneum, represent the major barrier components in the epidermis, which has turned out to be the only epithelial surface equipped with two structures that can be impaired in case of external or internal disturbance, such as in inflammatory conditions [5].

Skin inflammation can be the result of exposure to physical insults such as UV radiation, ionizing radiation, allergens, pathogens, or contact with chemical irritants [6].

Two of the most diffuse inflammatory cutaneous diseases are psoriasis vulgaris (PV) and atopic dermatitis (AD) [7]. The comprehension of the complex etiopathogenesis still needs to be completed, with the final aim of tailoring better therapeutic interventions [8].

PV is classified as an immune-mediated inflammatory disease due to the implication of both the innate and adaptive immune responses. The paradigm of a Th1-driven disease has been overcome by the discovery of the IL-23/Th-17 axis [9], in which interleukin (IL)-17A is a key proinflammatory cytokine. Its pathogenesis involves the combination of genetic susceptibility, altered immune response, and environmental factors [10].

AD is characterized by skin inflammation and barrier dysfunction, but its typical feature is represented by itch. The pathophysiology of AD is complex and involves abnormalities of the barrier and immune functions, but it is classified as a Th2-polarized disease, and common extrinsic AD patients express high levels of Th2 cytokines such as interleukin (IL)-4, IL-5, and IL-13 [11].

In the last decade, the relevance of barrier impairment has been emerging in the etiopathogenesis of both PV and AD. Regarding TJs, in PV, Cldn-1 is often completely absent, and a broader localization of ZO-1 is reported [12], whilst an overall reduction of all TJ components is demonstrated in AD [13]. The key difference is that AD lesions, but not PV plaques, are characterized by bacterial infections [5]. However, up until now, “there is an unmet need to better understand epidermal barrier regulation, not only as it applies to general skincare, but also to treatment of common skin conditions” [14].

In line with this scenario, Montero Vinchez et al. recently evaluated qualitative and quantitative skin alterations aimed at finding objective parameters to compare skin homeostasis and barrier function between healthy skin, psoriatic skin, and AD skin in a cross-sectional study [15]. The whole epidermal barrier was altered in both diseases, not only at lesional sites. In psoriatic plaques, TEWL, temperature, and erythema index were higher, and stratum corneum hydration was lower than in uninvolved psoriatic and healthy skin, while no differences in pH or elasticity were evident. Similar results were obtained in AD patients, with the difference that elasticity was also significantly lower at eczematous lesions than in healthy skin. Furthermore, temperature and TEWL values were suggested as useful parameters to predict disease severity, thus helping clinicians to select the most appropriate treatment. In the future, an evaluation of patients based on the different ongoing treatments that can differently affect the epidermal barrier will provide us with new insights and will help us to overcome one of the main limitations of the study.

In a parallel research study by the same group, a similar approach was used to investigate the impact of NB-UVB in a cross-sectional study considering psoriatic patients before and after receiving their first and the fifteenth phototherapy sessions [16]. In this case, the existing gap to fill was to determine which parameters could predict clinical improvement after NB-UVB phototherapy, i.e. a safe and affordable therapy for mild–moderate psoriasis that is able to block epidermal hyperproliferation and to exert an immunomodulatory effect. After phototherapy, epidermal barrier function and skin homeostasis were improved, and a cut-off point in the erythema corresponding to psoriatic plaques was identified after the first session. This finding could represent a useful landmark for physicians to select patients who would be more responsive to fifteen sessions as early as possible, thus reducing indirect costs and increasing the treatment’s cost-effectiveness.

Considering these recent results, we can conclude that a correlation between the quantitative parameters for the evaluation of the epidermal barrier and the expression/localization of TJ molecular components in psoriatic and AD patients would complete this complex tableau, providing a better understanding of TJ-mediated barrier impairment during skin inflammation.

## References

[1] Fu R., Jiang X., Li G., Zhu Y., Zhang H. (2021). Junctional Complexes in Epithelial Cells: Sentinels for Extracellular Insults and Intracellular Homeostasis. FEBS J.

[2] Green K.J., Jaiganesh A., Broussard J.A. (2019). Desmosomes: Essential Contributors to an Integrated Intercellular Junction Network. F1000Research.

[3] Meng W., Takeichi M. (2009). Adherens Junction: Molecular Architecture and Regulation. Cold Spring Harb. Perspect. Biol..

[4] Kirschner N., Brandner J.M. (2012). Barriers and More: Functions of Tight Junction Proteins in the Skin. Ann. N. Y. Acad. Sci..

[5] Beck L.A., Cork M.J., Amagai M., de Benedetto A., Kabashima K., Hamilton J.D., Rossi A.B. (2022). Type 2 Inflammation Contributes to Skin Barrier Dysfunction in Atopic Dermatitis. JID Innov..

[6] Bäsler K., Brandner J.M. (2017). Tight Junctions in Skin Inflammation. Pflugers Arch..

[7] Guttman-Yassky E., Krueger J.G. (2017). Atopic Dermatitis and Psoriasis: Two Different Immune Diseases or One Spectrum?. Curr. Opin. Immunol..

[8] Cannavò S.P., Guarneri F., Giuffrida R., Aragona E., Guarneri C. (2017). Evaluation of Cutaneous Surface Parameters in Psoriatic Patients. Skin Res. Technol..

[9] Prignano F., Donetti E. (2022). Looking at Interleukin-22 from a New Dermatological Perspective: From Epidermal Homeostasis to Its Role in Chronic Skin Diseases. Dermatology.

[10] Skutnik-Radziszewska A., Maciejczyk M., Flisiak I., Krahel J., Kołodziej U., Kotowska-Rodziewicz A., Klimiuk A., Zalewska A. (2020). Enhanced Inflammation and Nitrosative Stress in the Saliva and Plasma of Patients with Plaque Psoriasis. J. Clin. Med..

[11] Miraglia del Giudice M., Decimo F., Leonardi S., Maioello N., Amelio R., Capasso A., Capristo C., Capristo A.F. (2006). Immune Dysregulation in Atopic Dermatitis. Allergy Asthma Proc..

[12] Kirschner N., Poetzl C., von Den Driesch P., Wladykowski E., Moll I., Behne M.J., Brandner J.M. (2009). Alteration of Tight Junction Proteins Is an Early Event in Psoriasis: Putative Involvement of Proinflammatory Cytokines. Am. J. Pathol..

[13] de Benedetto A., Rafaels N.M., McGirt L.Y., Ivanov A.I., Georas S.N., Cheadle C., Berger A.E., Zhang K., Vidyasagar S., Yoshida T. (2011). Tight Junction Defects in Patients with Atopic Dermatitis. J. Allergy Clin. Immunol..

[14] de Benedetto A. (2021). Tight Junctions in the Skin: Still a Lot to Learn. BJD.

[15] Montero-Vilchez T., Segura-Fernández-Nogueras M.-V., Pérez-Rodríguez I., Soler-Gongora M., Martinez-Lopez A., Fernández-González A., Molina-Leyva A., Arias-Santiago S. (2021). Skin Barrier Function in Psoriasis and Atopic Dermatitis: Transepidermal Water Loss and Temperature as Useful Tools to Assess Disease Severity. J. Clin. Med..

[16] Montero-Vilchez T., Martinez-Lopez A., Sierra-Sanchez A., Soler-Gongora M., Jimenez-Mejias E., Molina-Leyva A., Buendia-Eisman A., Arias-Santiago S. (2021). Erythema Increase Predicts Psoriasis Improvement after Phototherapy. J. Clin. Med..

